# Laser Direct Writing of MnO_2_/Carbonized Carboxymethylcellulose-Based Composite as High-Performance Electrodes for Supercapacitors

**DOI:** 10.1021/acsomega.2c07350

**Published:** 2023-02-16

**Authors:** Kuan Ju, Yue Miao, Qi Li, Yabin Yan, Yang Gao

**Affiliations:** † Shanghai Key Laboratory of Intelligent Sensing and Detection Technology, School of Mechanical and Power Engineering, East China University of Science and Technology, Shanghai 200237, China; ‡ Wuhan National Laboratory for Optoelectronics, Huazhong University of Science & Technology, Wuhan 430074, Hubei, China

## Abstract

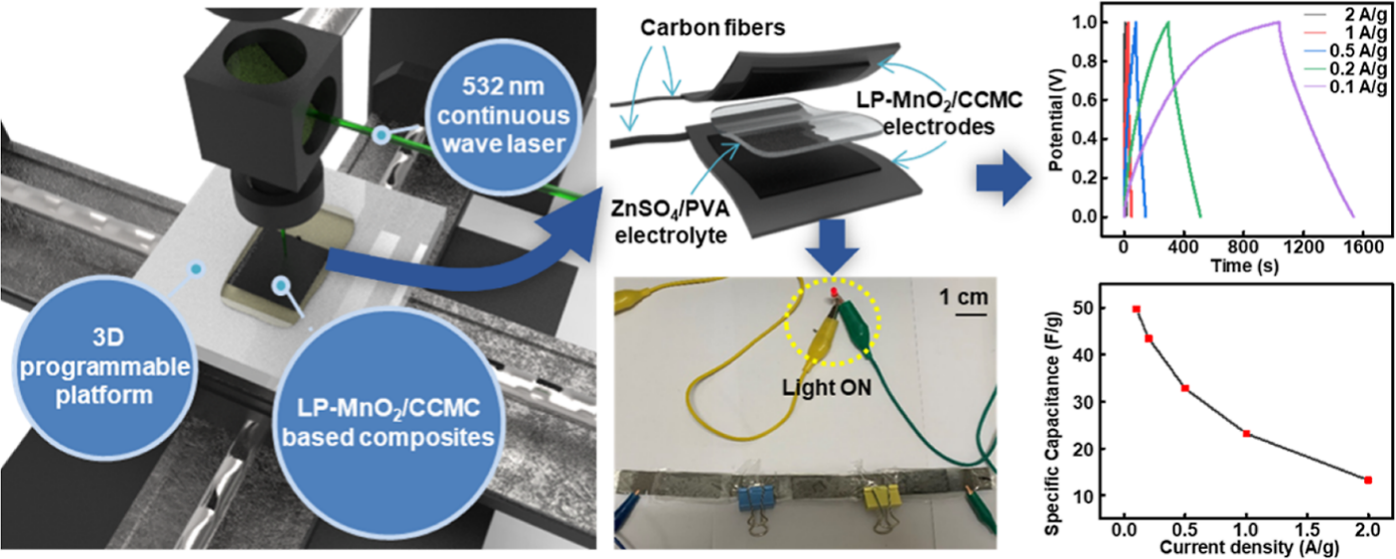

Manganese dioxide and its derivatives are widely used as promising electrode materials for supercapacitors. To achieve the environmentally friendly, simple, and effective material synthesis requirements, the laser direct writing method is utilized to pyrolyze the MnCO_3_/carboxymethylcellulose (CMC) precursors to MnO_2_/carbonized CMC (LP-MnO_2_/CCMC) in a one-step and mask-free way successfully. Here, CMC is utilized as the combustion-supporting agent to promote the conversion of MnCO_3_ into MnO_2_. The selected materials have the following advantages: (1) MnCO_3_ is soluble and can be converted into MnO_2_ with the promotion of a combustion-supporting agent. (2) CMC is an eco-friendly and soluble carbonaceous material, which is widely used as the precursor and combustion-supporting agent; (3) the redundant part of the MnCO_3_/CMC precursor can be removed by deionized water, which is simple and convenient. The different mass ratios of MnCO_3_ and CMC-induced LP-MnO_2_/CCMC(R1) and LP-MnO_2_/CCMC(R1/5) composites are investigated in the electrochemical performance toward electrodes, respectively. The LP-MnO_2_/CCMC(R1/5)-based electrode showed the high specific capacitance of 74.2 F/g (at the current density of 0.1 A/g) and good electrical durability for 1000 times charging–discharging cycles. Simultaneously, the sandwich-like supercapacitor which was assembled by LP-MnO_2_/CCMC(R1/5) electrodes presents the maximum specific capacitance of 49.7 F/g at the current density of 0.1 A/g. Moreover, the LP-MnO_2_/CCMC(R1/5)-based energy supply system is used to light a light-emitting diode, which demonstrates the great potential of LP-MnO_2_/CCMC(R1/5)-based supercapacitors for power devices.

## 1 Introduction

With the increase number of fast-charging mobile electronics and battery-powered vehicles, the high-powered energy storage tools were in tremendous demands. Supercapacitors have gained widespread attention due to their unique characteristics like high specific capacitance, high power density, and long cycle life.^1−4^ According to the energy storage mechanism, supercapacitors can be divided into two types: electrical double layered capacitor (EDLC) and pseudocapacitor. As for EDLCs, in the charging process, the polarized electrodes attracted the ions from the electrolyte solution to generate the electric double layers quickly, which led to the charging/discharging cycles in a short time. While for pseudocapacitors, the working mechanism could be ascribed to the Faradaic reactions occurring on the surface of conducting polymers and metal oxide based electrodes, causing the significant change of capacitance.^5^ There were a great deal of research studies that focused on these two types of supercapacitors, especially for the electrodes. The common electrode materials contained carbon-based nanomaterials, metal oxides, conducting polymers, and their nanocomposites, such as porous carbon,^6^ carbon nanotubes,^7^ graphene,^8^ MnO_*x*_,^9,10^ NiO,^11^ polyethylene dioxythiophene,^12^ and polyaniline,^13^ along with some novel materials like metal–organic frameworks,^14^ MXenes,^15^ metal nitrides, and so on.^16−18^ Among them, due to the high theoretical capacity and excellent electrochemical characterizations, the MnO_2_ and its derivatives were widespread used as a promising electrode material for supercapacitors.^19^

The redox reaction of MnO_4_^–^ or Mn^2+^ was universally employed to synthesize the MnO_2_ as well as its derivatives, and there are several methods to prepare MnO_2_-based electrodes, for instance, sol–gel processing, electrochemical deposition, coating, and inject-printing method.^20−22^ Typically, Chandra and coauthors demonstrated the MnO_2_ nanorod-based electrodes by the coating and magnetron sputtering procedures on silver porous-like substrate, which exhibited the high specific capacitance up to ∼796 F/g.^23^ ten Elshof and coworkers reported the δ-MnO_2_ nanosheets for a flexible micro-supercapacitor via the inject-printing method. The device delivered the volumetric capacitance of 2.4 F·cm^–3^ and high energy density of 1.8 × 10^–4^ Wh/cm^3^.^24^ Wang and coauthors presented the asymmetric supercapacitor with MnO_2_-based electrodes and Na_2_SO_4_ aqueous electrolyte because the MnO_2_ nanoparticles were generated by electrochemical deposition of KMnO_4_ in *N*-dimethylformamid solution.^25^ Furthermore, to enhance the electrochemical performance of MnO_2_, many efforts had developed not only on novel structures of MnO_2_ but also for the synergetic effect of MnO_2_ and the other conductive materials, such as activated carbon,^26^ hierarchical porous carbon,^27^ graphene,^28−30^ and nanotubes.^31,32^ Zhao and coworkers reported the MnO_2_ nanowire/CoAl-based hierarchical nanocomposite for high-performance supercapacitor, which displayed the high specific capacitance of 944 F/g (at the current density of 1 A/g), good stability, and excellent long-term cycling life.^33^ Li and coworkers developed the MnO_2_ nanoflakes/hierarchical porous carbon nanocomposites for supercapacitor electrodes by a two-step redox route.^34^ Although the aforementioned methods could fabricate the MnO_2_-based materials toward capacitance electrodes comprehensively, the following disadvantages were nonnegligible: (1) the large quantities usage of environmentally harmful reagents; (2) the complexity of the synthesis processes and long time-consuming, for example, the synthesis temperature of MnO_2_-based composites was up to 200 °C for 24 h.^35^ To address these shortcomings, an effective, compatible, and compact fabrication method for MnO_2_ synthesis was required.

Laser direct writing (LDW) could promote the redox reaction process of materials due to the distinctive photothermal effect. Because the laser beam led to the high temperature atmosphere for material, causing the obvious change of physical–chemical properties of the irradiated partial area. Thus, the LDW method was widely utilized to synthesis or change the characterizations of nanomaterials directly in ambient, gas, or liquid conditions, which was effective, scalable, and patternable. Over the past decade, the LDW-induced carbonaceous material, for instance, the laser induced graphene (LIG), the laser reduced graphene oxide (LRGO), and lignin-derived carbon were reported to generate the flexible electrodes or supercapacitors. Feng and coauthors demonstrated the LIG-based transparent supercapacitors in one-step with a high specific capacitance of 8.11 mF/cm^2^ and a volume capacitance density of 3.16 F/cm^3^ (0.05 mA/cm^2^).^36^ Cai and coworkers showed the LDW-induced LRGO–GO–LRGO interdigitated micro-supercapacitors, exhibiting the high capacitance of 12.5 mF/cm^2^ at the scan rate of 10 mV/s.^37^ Furthermore, LDW was also utilized to investigate the metallic-based materials for electrode synthesis. For instance, Lee et al. designed a flexible micro-supercapacitors with self-generate silver layers by laser-induced growth-sintering technique.^38^ Zhu et al. demonstrated LIG-decorated MONPs (M = Ti, Ni, Sn) electrodes by laser ablation in liquid phase conditions.^39^ Zhang et al. used the island-bridge structure to enhance the stretchability of micro-supercapacitor arrays and employed the LIG foam to generate a self-powered wireless wearable sensing platform.^2,3^ Cheng and coauthors demonstrated a new strategy to fabricate the functional circuits on 3D freeform surfaces by intense pulsed light-induced mass transfer of zinc nanoparticles.^40^ However, to our best knowledge, the study of the metallic-based supercapacitor generated by the LDW method was still in small quantity, particularly for MnO_2_-based composites due to the uncompleted redox reaction process and uncertainties of the secondary products.

Here, we used the LDW method to pyrolyze the MnCO_3_ and carboxymethylcellulose (CMC)-based precursors for electrode and supercapacitor. CMC was environmentally friendly, which could be easily dissolved in water and showed good material synergy performance with the other soluble materials. The MnCO_3_ was also water-soluble and could be oxidized to MnO_2_ by laser pyrolyzation process. As for MnCO_3_ and CMC composites, CMC was utilized as the combustion-supporting agent to promote the conversion of MnCO_3_ into MnO_2_, and the redundant part of the precursor would be removed by deionized (DI) water. The different mass ratios of MnCO_3_ and CMC (MnCO_3_/CMC = 1:1 and MnCO_3_/CMC = 1:5) were investigated to generate laser pyrolyzed MnO_2_ and CCMC composite [LP-MnO_2_/CCMC(R1) and LP-MnO_2_/CCMC(R1/5)], respectively. The LP-MnO_2_/CCMC(R1/5)-based electrodes with 1 M ZnSO_4_ electrolyte showed the highest specific capacitance of 74.2 F/g with the current density of 0.1 A/g. At the same time, the LP-MnO_2_/CCMC(R1/5)-based electrodes exhibited the electrical durability for 1000 times charge–discharge cycles. The LP-MnO_2_/CCMC(R1/5)-based supercapacitors with 1 M ZnSO_4_/1 M PVA electrolyte were also studied for the electrochemical performance, which showed the maximum specific capacitance of 49.7 F/g at the current density of 0.1 A/g. Moreover, the LP-MnO_2_/CCMC(R1/5)-based energy supply system was used to successfully light a LED, demonstrating the great potential of LP-MnO_2_/CCMC(R1/5)-based supercapacitors for power devices.

## 2 Results and Discussion

### 2.1 LDW Synthesis LP-MnO_2_/CCMC Composites

**Figure 1:**
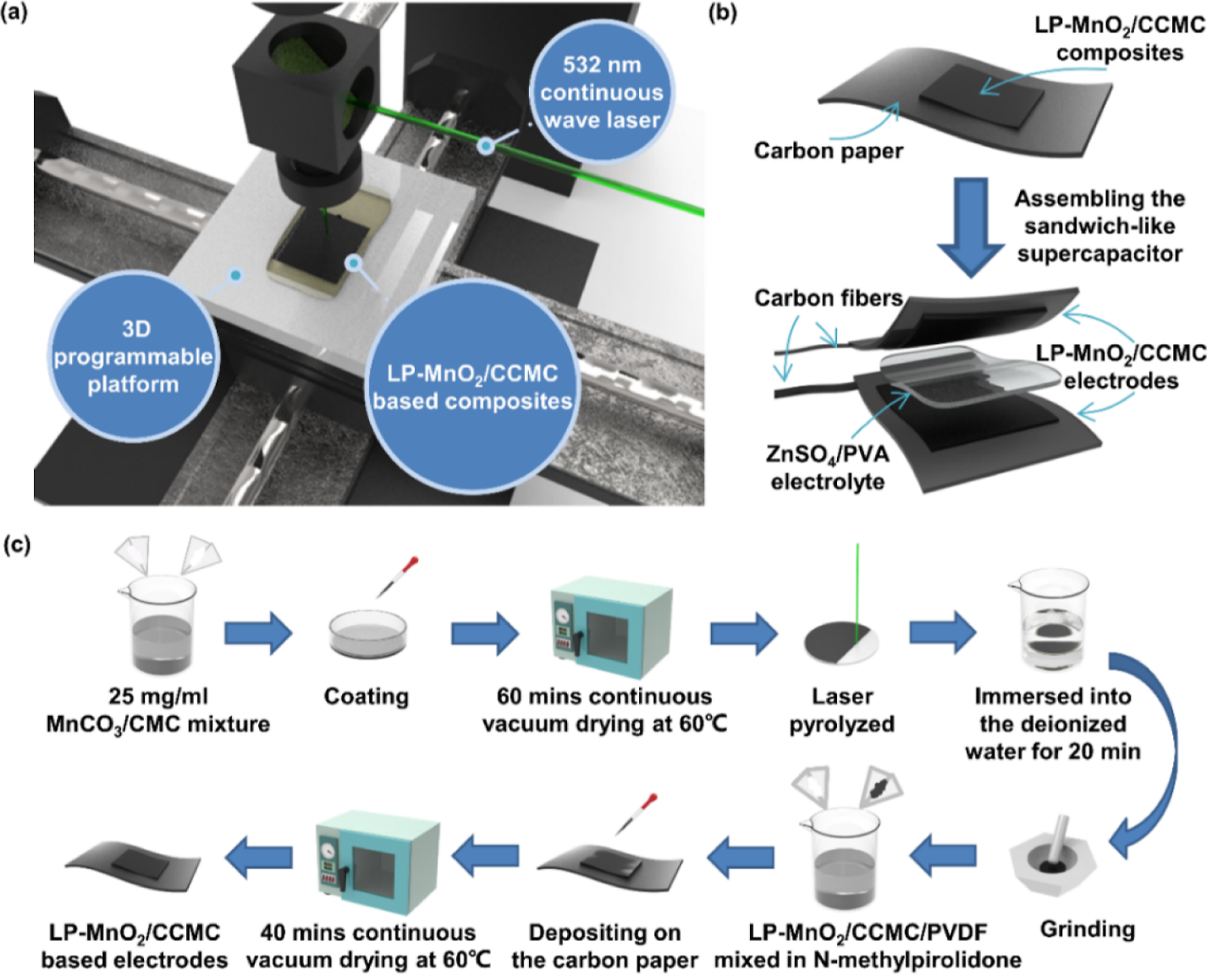
LP-MnO_2_/CCMC-based sandwich-like supercapacitor. (a) Schematic illustration exhibiting the LDW synthesis of the LP-MnO_2_/CCMC composites. (b) Schematic illustrating the components of the LP-MnO_2_/CCMC-based sandwich structure supercapacitor. (c) Schematic illustration of the fabrication procedures of the LP-MnO_2_/CCMC-based electrodes.

In this paper, the LDW method was proposed to fabricate LP-MnO_2_/CCMC-based electrodes and supercapacitors. Figure 1a showed the schematic illustration of the LDW system, which mainly contained the continuous wave laser (532 nm), a dichroic filter, and a programmable 3D translation stage. The laser power of 0.3 W and scanning speed of 2.5 mm/s were utilized to prepare LP-MnO_2_/CCMC-based composites, which were further assembled to the sandwich-like supercapacitor with a ZnSO_4_/PVA dielectric layer, as shown in Figure 1b. Figure 1c presented the detailed preparation process of the LP-MnO_2_/CCMC-based electrode.

**Figure 2:**
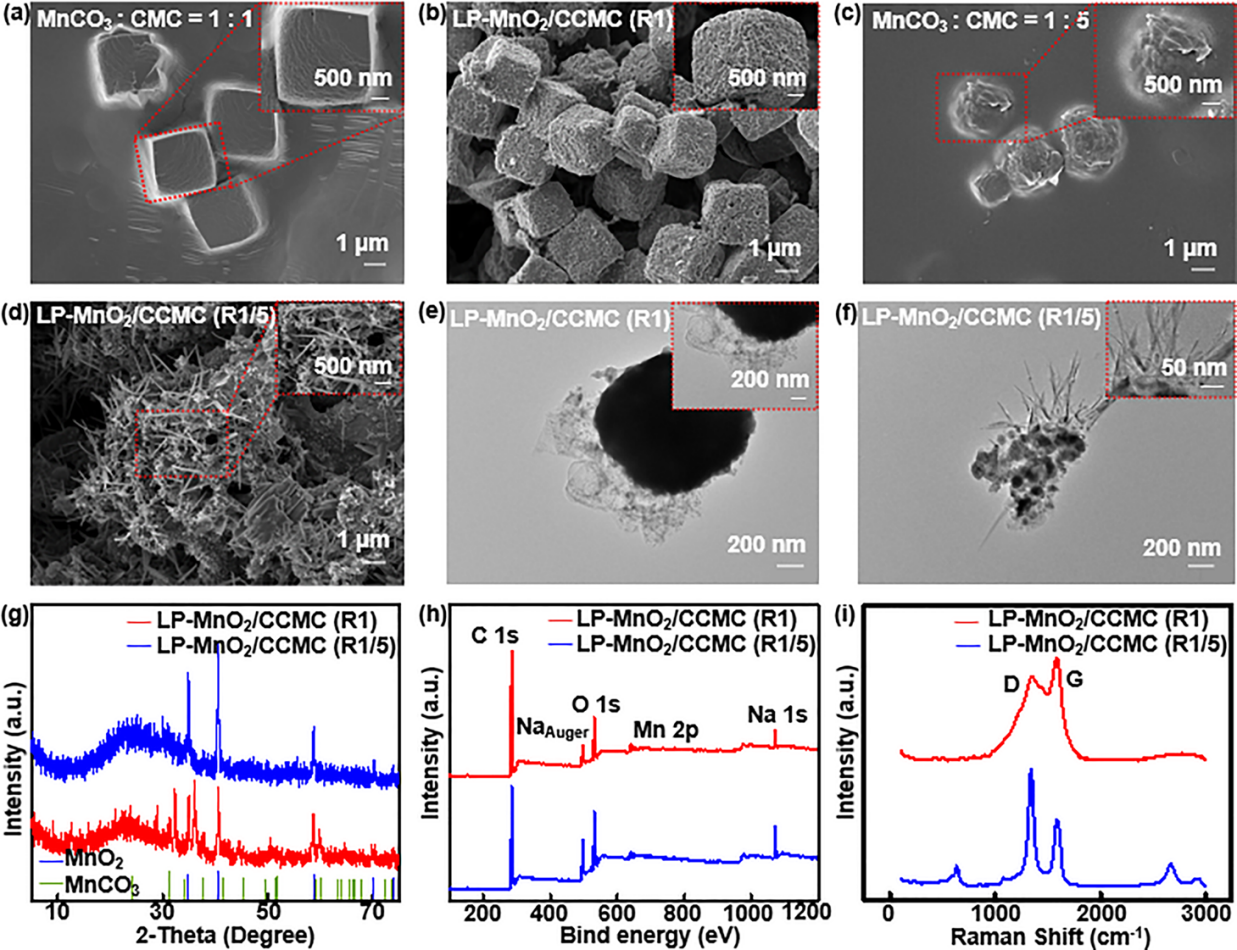
Characterization of the LP-MnO_2_/CCMC composites. (a,b) SEM images of the MnCO_3_/CMC precursors with a mass ratio of 1:1 and the LP-MnO_2_/CCMC composites (R1). (c,d) SEM images of the MnCO_3_/CMC precursors with a mass ratio of 1:5 and the LP-MnO_2_/CCMC composites (R1/5). (e,f) TEM images of the LP-MnO_2_/CCMC composites (R1) and LP-MnO_2_/CCMC composites (R1/5). (g) XRD spectra of the LP-MnO_2_/CCMC composites (R1) and LP-MnO_2_/CCMC composites (R1/5). (h) XPS spectra of the LP-MnO_2_/CCMC composites (R1) and LP-MnO_2_/CCMC composites (R1/5). (i) Raman spectra of the LP-MnO_2_/CCMC composites (R1) and LP-MnO_2_/CCMC composites (R1/5).

The morphologies and chemical compositions of LP-MnO_2_/CCMC composites are shown in Figure 2. Figure 2a,c demonstrated the scanning electron microscopy (SEM) images of MnCO_3_/CMC thin films with the mass ratios of 1:1 and 1:5. Figure 2b,d exhibited the LDW induced LP-MnO_2_/CCMC(R1) and LP-MnO_2_/CCMC(R1/5) composites by different mass ratios of MnCO_3_/CMC precursors, respectively. Compared with the MnCO_3_ without the LDW process (shown in Figure S1), the morphology of the LP-MnO_2_/CCMC(R1) composite presented the negligible structure change except for the much higher roughness of the particle surface. Meanwhile, the LP-MnO_2_/CCMC(R1/5) composite showed the significant morphologies changes with the increase of porous and needle-like nanostructures, which could be ascribed to the LDW-induced high throughput release of oxygen-containing gases. Herein, it should be noted that the CMC was employed as a combustion-supporting agent in the LDW process to promote the pyrolyzation of MnCO_3_. When the mass ratio of CMC in MnCO_3_/CMC composites was small (such as MnCO_3_/CMC = 1:1), the combustion-supporting effect could be quite weak and the morphology change of LP-MnO_2_/CCMC(R1) was indistinctive.

Figure 2e showed the transmission electron microscopy (TEM) image of LP-MnO_2_/CCMC(R1) composites, which had the large-area of layer shapes. Figure 2f illustrated the TEM image of significant needle-like nanostructures for LP-MnO_2_/CCMC(R1/5) composites. Consecutively, the EDS analysis mappings were utilized to investigate the elements distribution of LP-MnO_2_/CCMC(R1) and LP-MnO_2_/CCMC(R1/5) composites. As shown in Figure S2, both of the samples contained the C, Mn, Na, and O elements. Distinguishingly, the Na element was derived from the precursor of CMC. Figure S2a–e exhibited the elements mapping of LP-MnO_2_/CCMC(R1). In Figure S2c, the Mn mapping image was mainly concentrated in upper half regions, indicating an uneven distribution of elements, which mainly caused by the uncompleted pyrolyzation of MnCO_3_. Compared with the LP-MnO_2_/CCMC(R1) sample, the LP-MnO_2_/CCMC(R1/5) composites mapping exhibited the more uniform distribution of C, Mn, and O elements, illustrating the good carbonization and pyrolyzation of MnCO_3_ to MnO_2_ by the LDW method (Figure S2f–j).

For further investigation study, the chemical compositions of LP-MnO_2_/CCMC(R1) and LP-MnO_2_/CCMC(R1/5) were characterized by the X-ray diffractometer (XRD) test. As shown in Figure 2g, the crystal structure of LP-MnO_2_/CCMC(R1/5) was in high correlation with the face-centered cubic-based MnO_2_, which exhibited three typical peaks at 38.21, 45.26, and 59.82°. Inversely, although the LP-MnO_2_/CCMC(R1) spectrum also had three typical peaks nearby 38.21, 45.26, and 59.82°, the crystal structure of LP-MnO_2_/CCMC(R1) was more similar to the typical peaks of MnCO_3_, which addressed the incomplete pyrolyzation of the MnCO_3_/CMC precursors for the LDW process.

In addition, the X-ray photoelectron spectroscopy (XPS) spectrum of LP-MnO_2_/CCMC(R1) and LP-MnO_2_/CCMC(R1/5) are shown in Figure 2h. Both of the samples had elements of C, Mn, Na, and O. The typical peaks were occurred at 641.2, 652.8, 532.4, and 284.6 eV, representing the Mn 2p_3/2_, Mn 2p_1/2_, O 1s, and C 1s, respectively. Furthermore, the Mn 2p, O 1s, and C 1s spectra analysis of LP-MnO_2_/CCMC(R1) and LP-MnO_2_/CCMC(R1/5) are shown in Figure S3a–f. The Mn 2p spectra in Figure S3a,d could be fitted into Mn 2p_3/2_ and Mn 2p_1/2_, respectively. The O 1s spectra in Figure S3b,e showed the functional groups located nearby 531.88 and 535.7 eV, which could be ascribed to C=O and −OH, respectively. The C 1s spectra in Figure S3c,f could be disassembled into three peaks of 284.6, 285.2, and 289.3 eV, representing the functional groups of C=C, C–C, and C=O, respectively. Compared the C/Mn and C/O atomic ratios of LP-MnO_2_/CCMC(R1) and LP-MnO_2_/CCMC(R1/5) composites (Table S1), it was obviously discovered that the C/O atomic ratios were much higher in LP-MnO_2_/CCMC(R1/5) composite than LP-MnO_2_/CCMC(R1) sample, exhibiting the good pyrolyzation of LP-MnO_2_/CCMC(R1/5).

Raman spectroscopy was also adopted to identify the nanostructures and chemical components of LP-MnO_2_/CCMC(R1) and LP-MnO_2_/CCMC(R1/5) composites in Figure 2i. For the Raman spectra of LP-MnO_2_/CCMC(R1) composites, the typical peaks were located in 1387 and 1590 cm^–1^, which were attributed to the D- and G-bands. The typical peaks of Raman spectra for LP-MnO_2_/CCMC(R1/5) composites appeared at 1339 and 1587 cm^–1^. Noteworthily, the overlapping of the D- and G-bands illustrated the low crystallinity of the carbonaceous flakes. The *I*_D_/*I*_G_ of LP-MnO_2_/CCMC(R1) and LP-MnO_2_/CCMC(R1/5) composites were calculated as 5.515 and 1.642, respectively, which conveyed the much higher crystallinity and less defects of the LP-MnO_2_/CCMC(R1/5) synthesis material. Above the aforementioned results, the MnCO_3_/CMC precursors with a mass ratio of 1:5 were more suitable for preparing the LDW-induced LP-MnO_2_/CCMC(R1/5) electrodes, which would be used in the following sections.

### 2.2 Performance Study of LP-MnO_2_/CCMC-Based Electrodes

**Figure 3:**
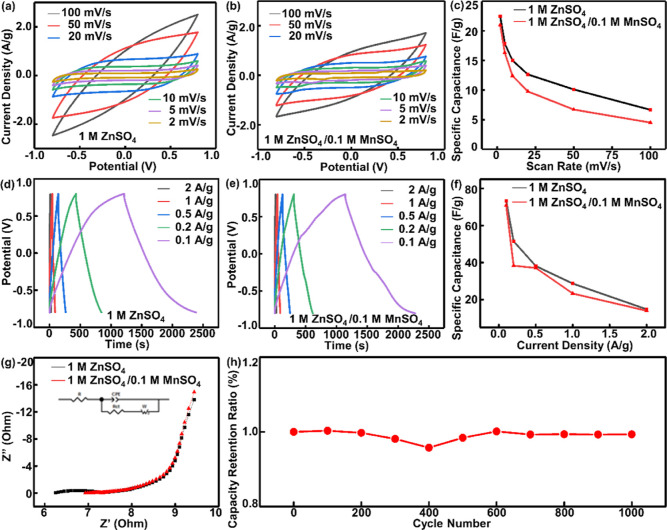
Electrical performance of the LP-MnO_2_/CCMC-based electrodes under the two-electrode system. (a,b) The CV curves of LP-MnO_2_/CCMC(R1/5) electrodes in 1 M ZnSO_4_ and 1 M ZnSO_4_/0.1 M MnSO_4_ electrolyte, respectively. (c) The specific capacitance vs scanning rate curves for LP-MnO_2_/CCMC(R1/5) electrodes in 1 M ZnSO_4_ and 1 M ZnSO_4_/0.1 M MnSO_4_ electrolyte, respectively. (d,e) GCD curves of LP-MnO_2_/CCMC(R1/5) electrodes in 1 M ZnSO_4_ and 1 M ZnSO_4_/0.1 M MnSO_4_ electrolyte with the current densities of 0.1–2 A/g. (f) Specific capacitance vs current density curves for LP-MnO_2_/CCMC(R1/5) electrodes in 1 M ZnSO_4_ and 1 M ZnSO_4_/0.1 M MnSO4 electrolyte. (g) EIS curves of LP-MnO_2_/CCMC(R1/5) electrodes in 1 M ZnSO_4_ and 1 M ZnSO_4_/0.1 M MnSO4 electrolyte. (h) Cyclic stability of the LP-MnO_2_/CCMC(R1/5) electrodes in 1 M ZnSO_4_ electrolyte.

To demonstrate the electrical performance of LP-MnO_2_/CCMC-based electrodes, the two-electrode method was utilized to investigate the characterizations of cyclic voltammetry (CV) and galvanostatic charge–discharge (GCD) tests for LP-MnO_2_/CCMC(R1/5)-based electrodes comprehensively. The potential window of test was set as −0.1 to 0.5 V and the scan speeds were used in 2–100 mV/s. Figure 3a,b displayed the CV curves of LP-MnO_2_/CCMC(R1/5) electrodes in 1 M ZnSO_4_ and 1 M ZnSO_4_/0.1 M MnSO_4_ electrolyte, respectively. With the increase of scanning speed, the closed shapes of the CV curve increased regularly to a symmetric rectangle, possessing the ideal capacitance performance of the LP-MnO_2_/CCMC(R1/5) electrodes. The scanning speed versus specific capacitance curves for 1 M ZnSO_4_ and 1 M ZnSO_4_/0.1 M MnSO_4_-based electrolyte are shown in Figure 3c. The specific capacitance of electrode in 1 M ZnSO_4_ electrolyte is higher than in 1 M ZnSO_4_/0.1 M MnSO_4_ electrolyte. When the scanning speed was 2 mV/s, the LP-MnO_2_/CCMC(R1/5) electrode with 1 M ZnSO_4_ electrolyte had the highest specific capacitance of 23.1 F/g.

Figure 3d,e shows the GCD curves of LP-MnO_2_/CCMC(R1/5) electrode in 1 M ZnSO_4_ electrolyte and 1 M ZnSO_4_/0.1 M MnSO_4_ electrolyte with the current densities of 0.1–2 A/g, respectively. For the charge/discharge tests in all current densities, the charge and discharge times of LP-MnO_2_/CCMC(R1/5) electrode were almost equivalent, presenting the reversible oxidation and reduction electrochemical process in charging and discharging cycles. The current density versus specific capacitance curves for 1 M ZnSO_4_ and 1 M ZnSO_4_/0.1 M MnSO_4_-based electrolyte are shown in Figure 3f. The specific capacitance of the LP-MnO_2_/CCMC(R1/5- based electrode in 1 M ZnSO_4_ electrolyte was slightly higher than that in 1 M ZnSO_4_/0.1 M MnSO_4_ electrolyte for all current density range. When the current density was 0.1 A/g, the specific capacitance of the electrode in 1 M ZnSO_4_ electrolyte was up to 74.2 F/g.

For investigating the diffusion of ions and the electrical conductivity of the electrodes, electrochemical impedance spectroscopy (EIS) tests were performed for LP-MnO_2_/CCMC(R1/5)-based electrodes in 1 M ZnSO_4_ electrolyte and 1 M ZnSO_4_/0.1 M MnSO_4_ electrolyte. Figure 3g depicts the EIS curve for the LP-MnO_2_/CCMC(R1/5)-based electrode and the fitted equivalent circuit. Herein, *R*, *R*_ct_, CPE, and *W*_o_ represented the electrolyte layer resistance, charge transfer resistance, constant-phase element, and finite-layer Warburg element, respectively. As shown in Table S2, the *R* and *R*_ct_ of LP-MnO_2_/CCMC(R1/5)-based electrodes in 1 M ZnSO_4_ electrolyte were smaller than in 1 M ZnSO_4_/0.1 M MnSO_4_ electrolyte. Moreover, the LP-MnO_2_/CCMC(R1/5) based electrodes in 1 M ZnSO_4_ electrolyte exhibited the stable electrical durability for 1000 times charge–discharge cycles, as shown in Figure 3h.

### 2.3 Performance Study of LP-MnO_2_/CCMC-Based Supercapacitor

**Figure 4:**
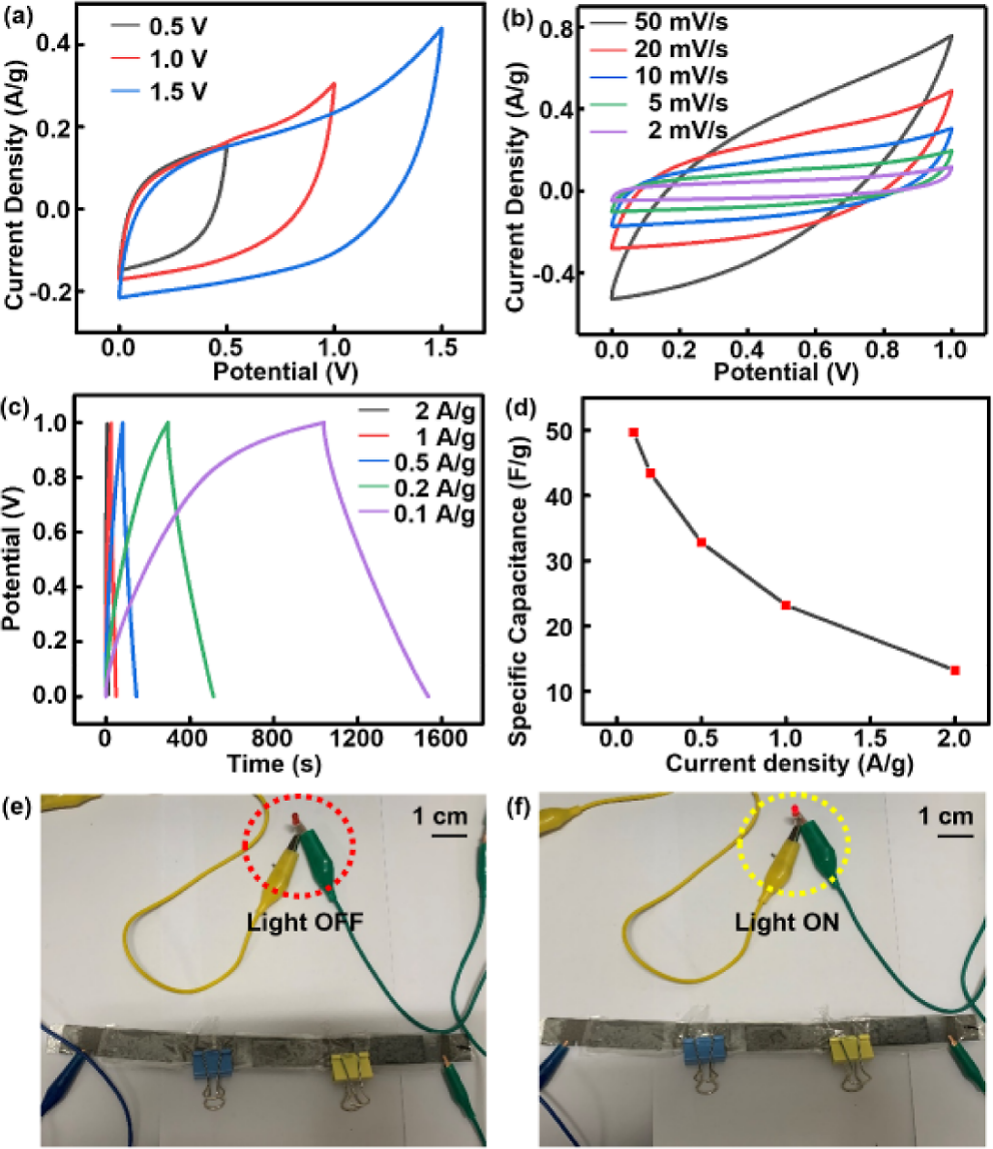
Electrical performance of the LP-MnO_2_/CCMC-based supercapacitor. (a) CV curves of LP-MnO_2_/CCMC-based supercapacitor at voltage windows of 0–0.5, 0–1.0, and 0–1.5 V, respectively. (b) CV curves of the LP-MnO_2_/CCMC-based supercapacitor at scan rates from 2 to 50 mV/s (c) GCD curves of LP-MnO_2_/CCMC-based supercapacitor with the current densities of 0.1–2 A/g. (d) Specific capacitance vs current density curves for the LP-MnO_2_/CCMC-based supercapacitor. (e,f) Photograph of three in series MnO_2_/CCMC supercapacitor used to power a LED.

Above the aforementioned electrical characterizations for different types of electrolytes, the ZnSO_4_-based electrolyte was more suitable for measurements due to the higher specific capacitance, which could be employed for LP-MnO_2_/CCMC-based supercapacitor. The symmetrical LP-MnO_2_/CCMC based supercapacitors were assembled with the LP-MnO_2_/CCMC(R1/5)-based electrodes and 1 M ZnSO_4_/1 M PVA gel electrolyte. Figure 4a showed the CV curves for potential windows of 0.0–0.5, 0.0–1.0, and 0.0–1.5 V at a scan rate of 10 mV/s. Under different potential windows, the CV curves were all closed in shapes of parallelograms without significant distortions. The further calculated results demonstrated that the LP-MnO_2_/CCMC(R1/5)-based supercapacitors had the largest specific capacitance of 11.77 F/g in the potential window of 0.0–1.0 V. Figure 4b shows the CV curves of LP-MnO_2_/CCMC(R1/5)-based supercapacitors at scan rates of 2–50 mV/s and the potential window of 0.0–1.0 V. It can be found that when the scan rate increased, the areal capacitance of the device increased regularly and the CV curves surrounded parallelograms maintained symmetrically, which illustrated the good capacitive behavior of LP-MnO_2_/CCMC(R1/5)-based supercapacitors.

The GCD curves for LP-MnO_2_/CCMC(R1/5)-based supercapacitors at current densities of 0.1–2 A/g are recorded in Figure 4c. Figure 4d showed the relationship between specific capacitance and current density of LP-MnO_2_/CCMC(R1/5)-based supercapacitors. The specific capacitance of the device was getting more and more larger with the continuous decrease of the current density. When the current density was 0.1 A/g, the specific capacitance of the device reached a maximum value of 49.7 F/g. Moreover, according to the calculation equation of energy density (*E*_d_), power density (*P*_d_), and coulombic efficiency (CE), as for LP-MnO_2_/CCMC-based electrodes, the maximum values of the *E*_d_, *P*_d_, and CE values were ascribed to 26.38 Wh/kg, 1.6 × 10^3^ W/kg, and 96.05%, respectively. For the LP-MnO_2_/CCMC-based supercapacitor, the *E*_d_, *P*_d_, and CE values were calculated as 6.90 Wh/kg, 1.0 × 10^3^ W/kg, and 47.83%, respectively. The Ragone plot of LP-MnO_2_/CCMC-based electrodes and supercapacitor with the other reported literature are shown in Figure S4 and Table S3.

### 2.4 Application of LP-MnO_2_/CCMC-Based Supercapacitor

To further study the energy supply performance of devices, three LP-MnO_2_/CCMC(R1/5)-based supercapacitors were in series connection for application tests. The series connection could increase the device test voltage, and the whole supercapacitor arrays were connected to a Chenhua electrochemical workstation for charging. Herein, the fully LP-MnO_2_/CCMC(R1/5) based energy supply system successfully lighted a LED, as shown in Figure 4e,f, which demonstrated the great potential of LP-MnO_2_/CCMC(R1/5)-based supercapacitors for power devices. Additionally, the electrical performances of the LP-MnO_2_/CCMC-based supercapacitor for stretching and bending tests are shown in Figure S5. In Figure S5a, the current density with bending angles of 3, 5, 7, and 10° is displayed, presenting the specific capacitances of 11.87, 8.97, 5.52, and 2.92 F/g. Figure S5b demonstrated the current density in stretching range of 1.6, 3.8, and 8.9%, which exhibited the minimum specific capacitance of 2.16 F/g. It could be concluded that with the increase of stretching range and bending angle, the specific capacitances of supercapacitor were in general decreasing, respectively.

## 3 Conclusions

In summary, the LP-MnO_2_/CCMC-based electrodes and supercapacitor were fabricated in a one-step and mask-free way by the LDW method. Notably, CMC was utilized as the combustion-supporting agent to promote the conversion of MnCO_3_ into MnO_2_. The different mass ratios (MnCO_3_/CMC = 1:1 and MnCO_3_/CMC = 1:5) induced LP-MnO_2_/CCMC [LP-MnO_2_/CCMC(R1) and LP-MnO_2_/CCMC(R1/5)] composites and different electrolytes (1 M ZnSO_4_ and 1 M ZnSO_4_/0.1 M MnSO_4_) were utilized to study the electrochemical performance toward electrodes, respectively. The LP-MnO_2_/CCMC(R1/5)-based electrode with 1 M ZnSO_4_ electrolyte showed the high specific capacitance of 74.2 F/g (at the current density of 0.1 A/g) and good electrical durability for 1000 times charge–discharge cycles. In addition, the sandwich-like supercapacitor assembled by LP-MnO_2_/CCMC(R1/5) electrodes and the 1 M ZnSO_4_/1 M PVA electrolyte, presenting the maximum specific capacitance of 49.7 F/g at the current density of 0.1 A/g. Moreover, the LP-MnO_2_/CCMC(R1/5)-based energy supply system were used to successfully light a LED, which demonstrated the great potential of LP-MnO_2_/CCMC(R1/5)-based supercapacitors for power devices.

## 4 Experimental Section

### 4.1 Materials

MnCO_3_ (AR), ZnSO_4_ (AR), and polyvinylidene fluoride (PVDF, AR) were purchased from Sinopharm Chemical Reagent Co., Ltd. CMC (AR), *N*-methylpyrrolidone (AR), and polyvinyl alcohol [(C_2_H_4_O)_*n*_, PVA, AR] were purchased from Shanghai Aladdin Biochemical Technology Co., Ltd. The above reagents were used as received without further purification.

### 4.2 LDW Synthesis of the LP-MnO_2_/CCMC-Based Electrodes

The mixture of MnCO_3_ and CMC solution with the mass ratio of 1:1 or 5:1 was prepared as precursors for the LDW process. Herein, the 25 mg/mL MnCO_3_/CMC mixture was poured into a Petri dish and dried at 60 °C to obtain the homogeneous thin film, respectively.

The 532 nm continuous wave laser (Verdi G10, Coherent Inc., beam diameter = 20 μm) and the 3D programmable platform (PSA150-11-X, Zolix Inc.) were utilized to pyrolyze the MnCO_3_/CMC thin film for LP-MnO_2_/CCMC(R1) and LP-MnO_2_/CCMC(R1/5) composites in the ambient environment. The laser power and scanning speed were maintained at 0.3 W and 2.5 mm/s in the entire LDW process unless the specific illustration. After the LDW procedures, the generated LP-MnO_2_/CCMC composites were immersed into the DI water for 20 min, which could remove the redundant the MnCO_3_/CMC composites thoroughly. The dried LP-MnO_2_/CCMC composites were grinded into powder and blended with PVDF in the mass ratio of 8:1. Then, dissolving the LP-MnO_2_/CCMC/PVDF mixed powder in *N*-methylpyrrolidone solution, the compound was uniformly depositing on the carbon paper in 1 cm × 1 cm. After drying the samples at 60 °C for 40 min, the LP-MnO_2_/CCMC-based electrodes were finally generated.

### 4.3 Assembly of the Sandwich-like LP-MnO_2_/CCMC-Based Supercapacitor

The ZnSO_4_/PVA electrolyte used for the LP-MnO_2_/CCMC-based supercapacitor was synthesized by dissolving 1 g of ZnSO_4_ and 1 g of PVA powder in 10 mL of DI water with magnetically stirring of 800 rpm at 100 °C. The sandwich-like LP-MnO_2_/CCMC-based supercapacitor was assembled with two layers of 1 cm × 1 cm LP-MnO_2_/CCMC electrodes and the homogeneous ZnSO_4_/PVA electrolyte.

### 4.4 Characterization of the LP-MnO_2_/CCMC Composites

The morphology of LP-MnO_2_/CCMC composites was demonstrated by field emission SEM (Hitachi, S4800) and TEM (JEM 2100, JEOL). The crystalline structures and chemical compositions of composites were investigated by XRD (Rigaku D/max2550VB/PC, Cu Kα1, λ = 1.541 Å), energy-dispersive X-ray spectroscopy, and XPS (ESCALAB 250, Thermo Fisher spectrometer). The molecular structure of LP-MnO_2_/CCMC samples was studied by Raman spectrum tests (Rigaku D/max 2550VB/PC, Cu Kα1, λ = 1.541 Å).

### 4.5 Electrochemical Characterization of the LP-MnO_2_/CCMC-Based Electrodes and Supercapacitor

All of the electrochemical characterization tests for LP-MnO_2_/CCMC-based electrodes and supercapacitors were performed by CHI660E electrochemical station (Shanghai Chenhua Instrument). The two-electrode method was utilized to investigate the electrochemical performance of LP-MnO_2_/CCMC-based electrodes and supercapacitors identically. Therein, the electrolyte for LP-MnO_2_/CCMC-based electrodes was composed of 1 M ZnSO_4_ aqueous solution or 1 M ZnSO_4_/0.1 M MnSO_4_ aqueous solution, respectively. The electrolyte for the LP-MnO_2_/CCMC-based supercapacitor consists of 1 M ZnSO_4_/1 M PVA gel, respectively.

As for LP-MnO_2_/CCMC-based electrodes and supercapacitor electrochemical characterizations, the CV tests were performed at scan rates of 2–100 mV/s; the GCD tests were measured at current densities of 0.1–2 A/g; the EIS tests were performed by 0.01–10^5^ Hz at open circuit potential.

Moreover, the capacitance (*C*_GCD_) of the supercapacitor at different current densities was obtained by the following eq 1
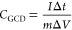
1where *I*, *m*, Δ*V*, and Δ*t* represent the current, the electrode mass, the potential window, and the discharge time, respectively.

The device’s capacitance (*C*_CV_) at different scan rates was obtained using the following eq 2
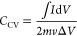
2where *I*, *m*, *v*, and Δ*V* ascribe to the current, the electrode mass, the scan rate, and the potential window, respectively.

Energy density (*E*_d_), power density (*P*_d_), and CE of the LP-MnO_2_/CCMC-based electrodes and supercapacitor were calculated by eqs 3–5 as follows
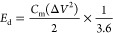
3where *C*_m_ and Δ*V* denote the gravimetric specific capacitance and potential window of supercapacitor, respectively.
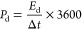
4where *E*_d_ and Δ*t* represent the energy density and the discharge time of supercapacitor, respectively.
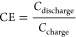
5

## Supporting Information Available

SEM images of the MnCO_3_; EDS analysis mappings of LP-MnO_2_/CCMC(R1) and LP-MnO_2_/CCMC(R1/5) composites; Mn 2p, O 1s, and C 1s XPS peak fitting diagram of LP-MnO_2_/CCMC(R1) and LP-MnO_2_/CCMC(R1/5); Ragone plot; electrical performance of the LP-MnO_2_/CCMC-based supercapacitor for the stretching and bending test; C/Mn and C/O atomic ratios; fitted electrical equivalent circuit values from EIS; and comparison of LP-MnO_2_/CCMC-based electrodes and supercapacitor with the other reported literature (PDF)
