# Supplementary material for: Laser Direct Writing of MnO2/Carbonized Carboxymethylcellulose-Based Composite as High-Performance Electrodes for Supercapacitors
Source: ACS Omega. 2023 Feb 16;8(8):7690–8. doi: 10.1021/acsomega.2c07350 (PMC9979346; doi:10.1021/acsomega.2c07350)
Supplement: Supplementary file 1 — ao2c07350_si_001.pdf [file ao2c07350_si_001.pdf]

Laser Direct Writing of MnO_2/Carbonized Carboxymethylcellulose based Composite as high-performance electrodes for supercapacitors

Kuan Ju[1], *Yue Miao*[1], *Qi Li* [1,*], *Yabin Yan* [1,*], *Yang Gao*[1,2,*]

1 Shanghai Key Laboratory of Intelligent Sensing and Detection Technology, School of Mechanical and Power Engineering, East China University of Science and Technology, Shanghai, 200237, China

2 Wuhan National Laboratory for Optoelectronics, Huazhong University of Science & Technology, Wuhan 430074, Hubei, China.

E-mail: liqi@ecust.edu.cn (Q. Li); yanyabin@ecust.edu.cn (Y. Yan); yanggao@ecust.edu.cn (Y. Gao)

KEYWORDS

Laser direct writing; Manganese oxide composite; Carboxymethylcellulose; Supercapacitor

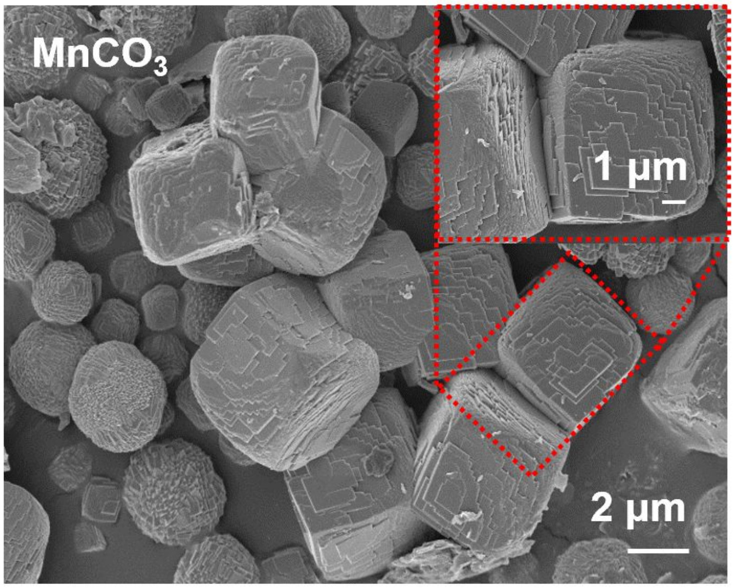

Figure S1. SEM images of the $MnCO_3$.

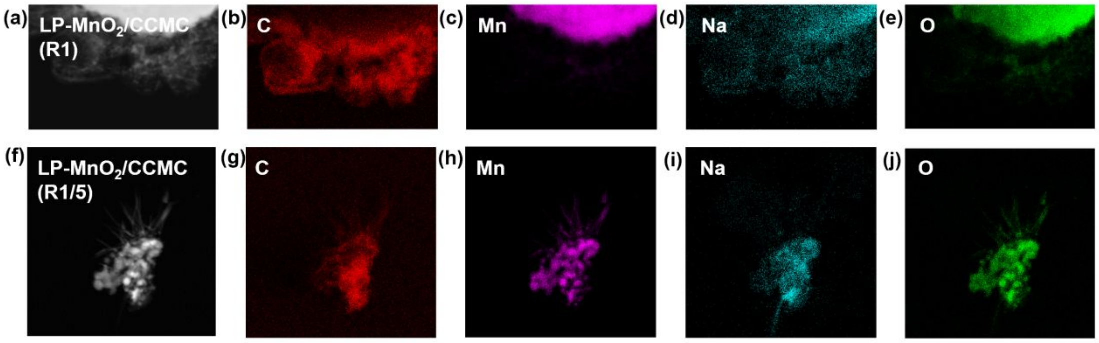

Figure S2. EDS analysis mappings of LP-MnO_2/CCMC(R1) and LP-MnO_2/CCMC(R1/5) composites. (a-e) C, Mn, Na, and O elements mapping of LP-MnO_2/CCMC (R1). (f-j) C, Mn, Na, and O elements mapping of LP-MnO_2/CCMC (R1/5).

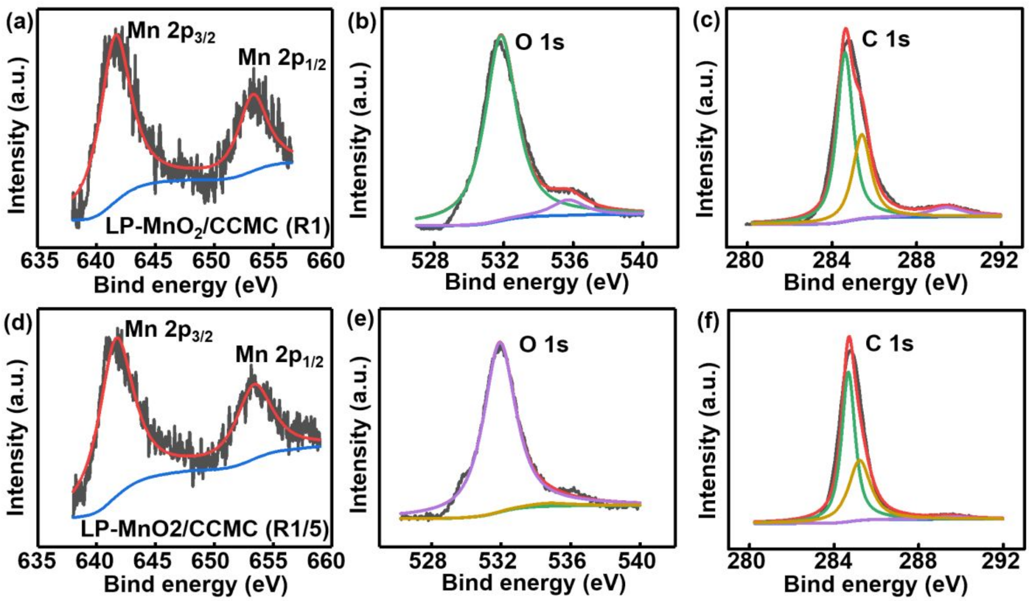

Figure S3. Mn 2p (a) O 1s (b) and C 1s (c) XPS peak fitting diagram of LP-MnO_2/CCMC(R1).

Mn 2p (d) O 1s (e) and C 1s (f) XPS peak fitting diagram of LP-MnO_2/CCMC(R1/5).

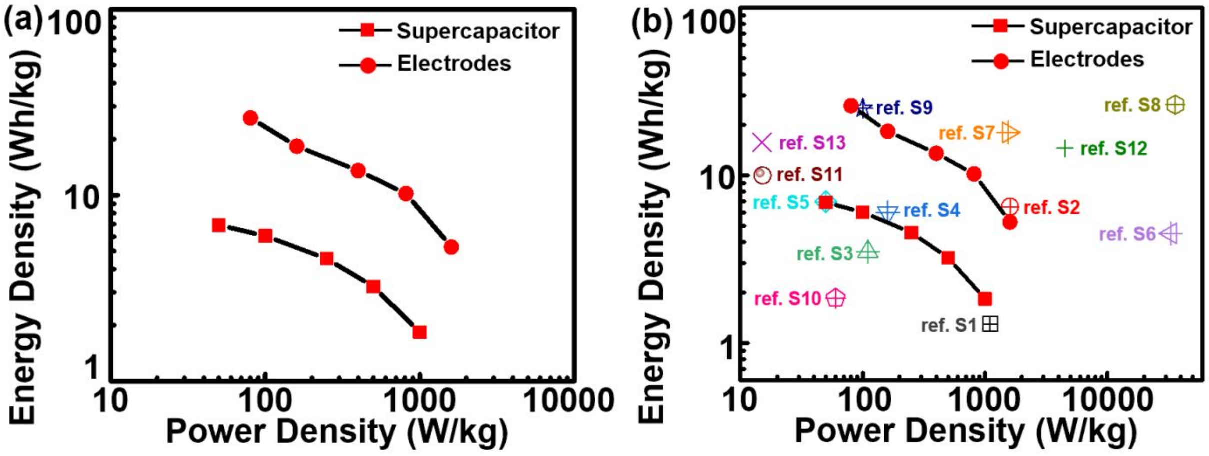

Figure S4. Ragone plot of (a) LP-MnO_2/CCMC based electrodes and supercapacitor and (b) the comparison with the other reported literature.

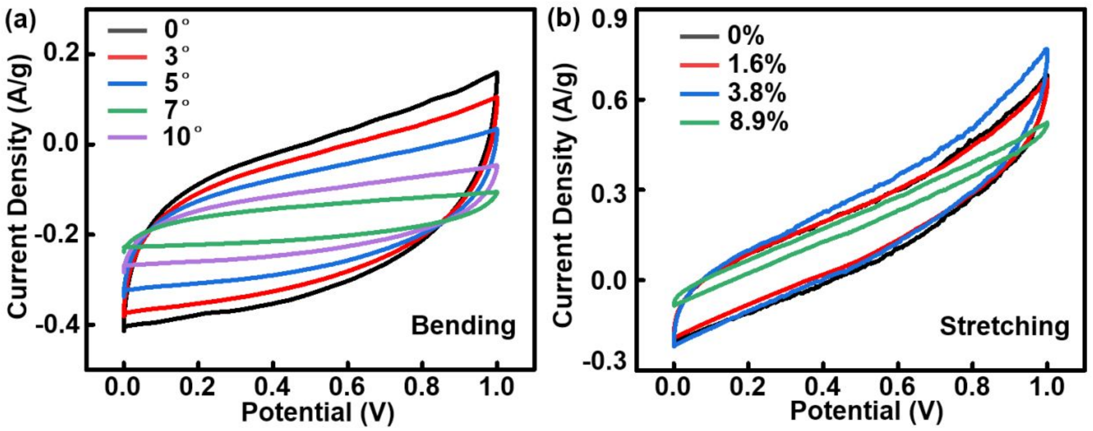

Figure S5. Electrical performance of the LP-MnO_2/CCMC based supercapacitor for stretching and bending test. (a) CV curves of LP-MnO_2/CCMC based supercapacitor at the bending angle of 3˚, 5˚, 7˚ and 10˚ respectively. (b) CV curves of LP-MnO_2/CCMC based supercapacitor at the strain of 1.6%, 3.8% and 8.9%, respectively.

Table S1. C/Mn and C/O atomic ratios of LP-MnO_2/CCMC(R1) and LP-MnO_2/CCMC(R1/5) composites.

	**LP-MnO_2/CCMC (R1/5)**	**LP-MnO_2/CCMC (R1)**
**O/C**	**0.2003**	**0.3356**
**Mn/C**	**0.0153**	**0.0169**

Table S2. Fitted electrical equivalent circuit values from EIS of LP-MnO_2/CCMC(R1/5) based electrodes in 1 M $ZnSO_4$ and 1 M $ZnSO_4$/0.1 M $MnSO_4$ electrolyte.

Sample	R/Ω	R_{ct}/Ω	CPE	W_o
**1 M $ZnSO_4$/ 0 M $MnSO_4$**	**7.44**	**2.13**	**0.18**	**0.078**
**1 M $ZnSO_4$/ 0.1 M $MnSO_4$**	**8.44**	**3.56**	**0.33**	**0.077**

Table S3. The comparison of LP-MnO_2/CCMC based electrodes and supercapacitor with the other reported literature.

Materials	Energy density (Wh/kg)	Power density (W/kg)	Electrolyte	Ref.
Nanostructured Mn_3O_4	4.2	1.1×10^3	1 M Na_2SO_4	[S1]
Poly(ionic liquid)-Modified Graphene	6.5	2.4×10^3	EMIM-NTf_2	[S2]
CMG//MnO_2/CMG	3.5	1.05×10^3	1 M Na_2SO_4	[S3]
MnO_2-CNFMs	6.0	0.16×10^3	1 M Na_2SO_4	[S4]
rGO/MnO_x	6.96	2.0×10^3	6 mol/L KOH	[S5]
Au-MnO_2/CNT	4.5	33×10^3	0.1 M Na_2SO_4	[S6]
Mn-SnS_2-GA	18.02	1.55×10^3	6 mol/L KOH	[S7]
α-MnO_2 nanorods/Ag/AAO	26.4	36×10^3	1 M Na_2SO_4	[S8]
MnO_2/graphene	25.2	0.1×10^3	1M Na_2SO_4	[S9]
MnO_2// MnO_2-Au	1.85	0.06×10^3	0.1 M Na_2SO_4	[S10]
ZnO@LIG	10	15.17	PVA/KCl	[S11]
ZnP@LIG	14.5	4.5×10^3	PVA/KCl	[S12]
Co_3O_4@LIG	15.8	15	/	[S13]
MnO_2/CCMC	26.38/6.9	1.6×10^3/1.0×10^3	1 M $ZnSO_4$	This work

Reference

[S1] Dubal, D. P.; Jagadale, A. D.; Lokhande, C. D., Big as well as light weight portable, Mn_3O_4 based symmetric supercapacitive devices: Fabrication, performance evaluation and demonstration. *Electrochim. Acta* ***2012***, *80*, 160-170.

[S2] Kim, T. Y.; Lee, H. W.; Stoller, M.; Dreyer, D. R.; Bielawski, C. W.; Ruoff, R. S.; Suh, K. S., High-Performance Supercapacitors Based on Poly (ionic liquid)-Modified Graphene Electrodes. *ACS Nano* **2011**, *5* (1), 436-442.

[S3] Choi, B. G.; Yang, M.; Hong, W. H.; Choi, J. W.; Huh, Y. S., 3D Macroporous Graphene Frameworks for Supercapacitors with High Energy and Power Densities. *ACS Nano* **2012**, *6* (5), 4020-4028.

[S4] Youe, W.-J.; Kim, S. J.; Lee, S.-M.; Chun, S.-J.; Kang, J.; Kim, Y. S., MnO_2-deposited lignin-based carbon nanofiber mats for application as electrodes in symmetric pseudocapacitors. *Int. J. Biol. Macromol.* ***2018***, *112*, 943-950.

[S5] Luan, Z.; Tian, Y.; Gai, L.; Jiang, H.; Guo, X.; Yang, Y., Environment-benign synthesis of rGO/MnO_x nanocomposites with superior electrochemical performance for supercapacitors. *J. Alloys Compd.* **2017**, *729*, 9-18.

[S6] Reddy, A. L. M.; Shaijumon, M. M.; Gowda, S. R.; Ajayan, P. M., Multisegmented Au-MnO_2/Carbon Nanotube Hybrid Coaxial Arrays for High-Power Supercapacitor Applications. *J. Phys. Chem. C* **2010**, *114* (1), 658-663.

[S7] Chu, H.; Zhang, F.; Pei, L.; Cui, Z.; Shen, J.; Ye, M., Ni, Co and Mn doped SnS_2-graphene aerogels for supercapacitors. *J. Alloys Compd.* ***2018***, *767,* 583-591.

[S8] Kumar, A.; Sanger, A.; Kumar, A.; Mishra, Y. K.; Chandra, R., Performance of High Energy Density Symmetric Supercapacitor Based on Sputtered MnO_2 Nanorods. *Chemistryselect* ***2016***, *1*

(13), 3885-3891.

[S9] Cao, J.; Wang, Y.; Zhou, Y.; Ouyang, J.-H.; Jia, D.; Guo, L., High voltage asymmetric supercapacitor based on MnO_2 and graphene electrodes. *J. Electroanal. Chem.* ***2013**, 689*, 201-206.

[S10] Liu, C.-C.; Tsai, D.-S.; Chung, W.-H.; Li, K.-W.; Lee, K.-Y.; Huang, Y.-S., Electrochemical micro-capacitors of patterned electrodes loaded with manganese oxide and carbon nanotubes. *J. Power Sources* **2011**, *196* (13), 5761-5768.

[S11] Peng, Z.; Jia, J.; Ding, H.; Yu, H.; Shen, Y.; Zhang, J.; Tao, W.; Zhang, C.; Wang, J.; Cheng, H., High-energy all-in-one micro-supercapacitors based on ZnO mesoporous nanosheet-decorated laser-induced porous graphene foams. *J. Mater. Res.* **2021,** *36* (9), 1927-1936.

[S12] Zhang, C.; Peng, Z.; Huang, C.; Zhang, B.; Xing, C.; Chen, H.; Cheng, H.; Wang, J.; Tang, S., High-energy all-in-one stretchable micro-supercapacitor arrays based on 3D laser-induced graphene foams decorated with mesoporous ZnP nanosheets for self-powered stretchable systems. *Nano Energy* **2021,** *81*, 105609.

[S13] Ding, X.; Liu, R.; Zhao, J.; Hu, J.; Wu, J.; Zhang, C.; Lin, J., In situ formation of Co3O4 nanocrystals embedded in laser-induced graphene foam for high-energy flexible micro-supercapacitors. *Dalton Transactions* **2022,** *51* (7), 2846-2854.
